# Researcher profiling systems: fostering collaboration on a regional medical campus and clinical and translational science award institution

**DOI:** 10.5195/jmla.2023.1622

**Published:** 2023-10-02

**Authors:** Ashley Zeidler, Wes Rood

**Affiliations:** 1 azeidler@mcw.edu, Scholarly Communications Librarian, Medical College of Wisconsin Libraries, Milwaukee, WI.; 2 wrood@mcw.edu, Software Engineer, CTSI Center for Biomedical Informatics, Medical College of Wisconsin, Milwaukee, WI.

**Keywords:** Academic Research, Biomedical Research, Clinical Research, Librarian Collaboration, Curriculum Vitae

## Abstract

The Faculty Collaboration Database (FCD) is a researcher profiling system that promotes collaboration for the Medical College of Wisconsin and its research partners through the Clinical and Translational Science Institute of Southeast Wisconsin (CTSI). Those institutions include Children's Wisconsin, Froedtert Hospital, Marquette University, Milwaukee School of Engineering, University of Wisconsin Milwaukee, Milwaukee VA Medical Center, and Versiti.

The Faculty Collaboration Database (FCD) (https://fcd.mcw.edu/) is used to foster collaboration between researchers within a regional biomedical research cohort and beyond. Researchers promote their clinical, research, and methodological expertise on publicly available profiles. It was created and is maintained by the research software engineering team, led by Wes Rood, in the CTSI Center for Biomedical Informatics (CBMI), with librarians providing training, end user support, and promotion.

Due to the success of the initial profiling system created in 2008 that featured only publication lists, various groups on campus have advocated for enhancements, such as a mode to enter curricula vitae (CV) in the institution's standard CV format, a National Institute of Health (NIH) Biosketch generator, and the ability to indicate willingness to serve as a research mentor.

The web-based system was built in PHP and data is organized in an Oracle relational database. Faculty profiles utilize an Application Programming Interface (API) from MCW's human resources (HR) system to initially populate the profiles. Additional APIs from Scopus and PubMed are used to fetch publications through author-based searches. Publication abstracts and Medical Subject Headings (MeSH) terms are collected from the PubMed API. Everything in the researcher profile is fully searchable – including content in the CVs, publication abstracts, and terms.

Publications, MeSH terms, and basic profile demographics are available publicly to anyone visiting the site, and CVs can be made public or private at the individual item level. Because much of the information on profiles is public, and to facilitate collaboration throughout the global research community, there is a contact form available on each profile to help make that first connection.

Researchers can use content in their profiles and CVs to generate NIH Biosketches, and the Biosketch format is kept current. Researchers can assign designees to maintain their profiles, publication lists, CVs and Biosketches. A new enhancement allows anyone with an account to generate publication reports for a department, division, or center, thus creating an efficient way to gather this information from the author-verified lists. These reports incorporate Journal Impact Factor and Top Tier classification data that is curated by MCW librarians.

The FCD is well used at MCW. Of the 3074 active profiles created for MCW researchers, 77% have logged into the profiling system at least once, almost half have entered information into their CVs, and there are 539 Biosketches in the system. Between 2020-2021, over 18300 articles have been added to the profiles and 1285 messages were sent through the contact feature.

Efforts are underway to promote the publication report feature from the FCD, as departmental administrators are interested in this feature to measure faculty research output and impact. MCW Libraries has implemented a research impact challenge (https://mcw.libguides.com/research-impact-challenge) that consists of five tasks that will not only help researchers raise the visibility and impact of their research but also ensure that FCD profiles remain up to date.

